# Two-Component Direct Fluorescent-Antibody Assay for Rapid Identification of *Bacillus anthracis*

**DOI:** 10.3201/eid0810.020392

**Published:** 2002-10

**Authors:** Barun K. De, Sandra L. Bragg, Gary N. Sanden, Kathy E. Wilson, Lois A. Diem, Chung K. Marston, Alex R. Hoffmaster, Gwen A. Barnet, Robbin S. Weyant, Teresa G. Abshire, John W. Ezzell, Tanja Popovic

**Affiliations:** * Centers for Disease Control and Prevention, Atlanta, Georgia, USA; † U.S. Army Medical Institute of Infectious Diseases, Fort Detrick, Maryland, USA

**Keywords:** *Bacillus anthracis*, DFA

## Abstract

A two-component direct fluorescent-antibody (DFA) assay, using fluorescein-labeled monoclonal antibodies specific to the *Bacillus anthracis* cell wall (CW-DFA) and capsule (CAP-DFA) antigens, was evaluated and validated for rapid identification of *B. anthracis.* We analyzed 230 *B. anthracis* isolates; 228 and 229 were positive by CW-DFA and CAP-DFA assays, respectively. We also tested 56 non–*B. anthracis* strains; 10 *B. cereus* and 2 *B. thuringiensis* were positive by the CW-DFA assay, and 1 *B. megaterium* strain was positive by CAP-DFA. Analysis of the combined DFA results identified 227 of 230 *B. anthracis* isolates; all 56 strains of the other *Bacillus* spp. were negative. Both DFA assays tested positive on 14 of 26 clinical specimens from the 2001 anthrax outbreak investigation. The two-component DFA assay is a sensitive, specific, and rapid confirmatory test for *B. anthracis* in cultures and may be useful directly on clinical specimens.

The potential use of *Bacillus anthracis* as a biological weapon has long been recognized (1–5). Recently, the profound impact of *B. anthracis* on public health was demonstrated during the bioterrorism-related anthrax outbreak in the United States (6). Rapid diagnosis played an important role during the outbreak and aided in implementing appropriate public health measures in a timely manner. Although several standard microbiologic assays are available to identify *B. anthracis* (7)*,* they primarily lack timeliness in producing results.

Earlier studies demonstrated the advantages of immunofluorescence assays, based on polyclonal antibodies to *B. anthracis* cell-surface antigens, for identifying *B. anthracis* isolates (8) and directly evaluating clinical specimens from infected guinea pigs (9). However, the limitations of polyclonal antibodies, such as the problem of cross-reactivity with closely related *Bacillus* species known as *B. cereus* complex (10), were also apparent. Over the past decade, monoclonal antibodies specific to the *B. anthracis* cell wall polysaccharide antigen were shown to be useful in diagnosing *B. anthracis* infection (11,12). Vegetative *B. anthracis* cells constitutively express the galactose/N-acetylglucosamine polysaccharide cell wall antigen (13,14). In addition, during infection or growth in nutrient-rich media in an elevated CO_2_ environment, *B. anthracis* cells produce a poly-γ-D-glutamic acid capsule, which is synthesized by the products of genes located on the pXO2 plasmid (15). In this study, we have evaluated and validated a two-component direct fluorescent-antibody (DFA) assay, using the monoclonal immunoglobulin (Ig) M antibody EAII-6G6-2-3 against the cell wall polysaccharide antigen (CW) (12) and the monoclonal IgG antibody FDF-1B9 against the capsule antigen (CAP) (16) for rapid identification of *B. anthracis*. In addition to use on isolates, this rapid DFA assay was applied successfully to detect *B. anthracis* directly in clinical specimens from several patients with laboratory-confirmed inhalational anthrax during the 2001 bioterrorism-associated anthrax outbreak in the United States (6,17).

## Materials and Methods

### Bacterial Isolates

#### *B. anthracis* Isolates (n=230)

 Eighty-one *B. anthracis* isolates from different sources (human, animal, and environmental) representing broad geographic and temporal (1939–1997) diversity were selected from culture collections at the Meningitis and Special Pathogens Branch, Centers for Disease Control and Prevention, Atlanta, Georgia. Six of these isolates were free of pXO1 or pXO2 plasmids. An additional 149 *B. anthracis* isolates, obtained from powders (n=4), 10 patients (n=20), and environmental sources (n=125) during the investigation of the U.S. bioterrorism-associated anthrax outbreak from October 5 to December 21, 2001, were included.

####  Other *Bacillus* spp. (n=56)

 Five closely related *Bacillus* species—*B. cereus* (n=23), *B. megaterium* (n=11), *B. subtilis* (n=9), *B. thuringiensis* (n=12), and *B. mycoides* (n=1)—were selected to test the specificity of the DFA assays. Most *B. cereus* isolates (n=20) were from different sources (environmental, food, human, and animal) representing broad geographic and temporal (1957–2000) diversity.

####  Control Strains (n=2)

*B. anthracis* Pasteur (ATCC 4229) and *B. cereus* (ATCC 14579) were used as positive and negative controls, respectively, for both CW and CAP DFA assays. The control strains were stored at 4°C as spore suspensions in water. All other strains were kept at –70°C as spore suspensions in water or in 2.5% heart infusion broth (HIB) containing 20% glycerol. All strains were identified by standard microbiologic procedures (7), and confirmatory identification of *B. anthracis* strains was performed according to the Laboratory Response Network testing algorithm (5) using a battery of tests including the DFA assay described in this study.

### Clinical Specimens

Twenty-six clinical specimens, including aerobic and anaerobic blood cultures (n=11), various body fluids (n=6), pleural fluids (n=4), lung tissues (n=3), and lymph nodes (n=2), were collected from seven patients with laboratory-confirmed inhalational anthrax from October through December 2001 (6,17,18).

### Preparation of Fluorescein-Antibody Conjugates

Two monoclonal antibodies, EAII-6G6-2-3 (12) and FDF-1B9 (16), were purified by HiTrap SP Gradifrac cation exchange chromatography (Pharmacia, Peapack, NJ) to homogeneity and conjugated to fluorescein isothiocyanate (FITC), according to a standard protocol (Molecular Probes, Eugene, OR). The anti-cell wall (anti-CW FITC) and anti-capsule (anti-CAP FITC) conjugates were lyophilized in HEPES buffer (0.05 M HEPES, pH 7.0, 0.10% glycine, 0.01 M d-sorbitol, 0.15 M KCl, and 5% d-trehalose) containing 1% bovine serum albumin (Cohn Fraction V) (Sigma Chemical Co., St. Louis, MO). The working antibody solutions (50 µg/mL) were prepared in 50% glycerol in water and stored at –20°C or 4°C.

### Preparation of Cell Suspensions for DFA Assays

 Vegetative Cells for the CW-DFA Assay

 For each control and test strain, fresh vegetative cells were grown by plating stock spore suspension (1 µL) on trypticase soy agar with 5% sheep blood (SBA) (BBL Microbiology Systems, Cockeysville, MD) and incubating aerobically overnight at 37°C. The cell suspensions were prepared by suspending one loop (1 µL) of the SBA culture in 100 μL of 10 mM phosphate-buffered saline/0.3% Tween 20, pH 7.2 (PBST) and adjusting the concentration to ~10^7^ cells/mL (equivalent to a 0.5 McFarland standard).

####  Encapsulated Cells for the CAP-DFA Assay

For each control and test strain, encapsulated cells were grown by transferring an overnight growth of fresh vegetative cells (~10^7^ cells) into either 450 µL of defibrinated horse blood (Lampire Biological Labs, Pipersville, PA) or 2.5% HIB supplemented with 50% inactivated horse serum (Sigma) and 0.8% sodium bicarbonate and incubating at 37°C for 3 h.

####  Clinical Specimens

 For liquid specimens, ~90 µL of each specimen was diluted with 10 vol of PBST; the cells were recovered by centrifugation (14, 000 X *g* for 3 min). After removal of supernatant, the cells were suspended in 90 µL of the residual supernatant. Solid tissues (e.g., lymph nodes, lung tissues) were homogenized with a small disposable tissue grinder (Kendall Co., Mansfield, MA) in 100–250 µL of HIB. Forty-five microliters of the homogenates or cell suspensions was used directly in the DFA assays.

### CW- and CAP-DFA Assays

To evaluate the sensitivity and specificity of both DFA assays, 45-µL cell suspensions were mixed with 5 µL of anti-CW FITC or anti-CAP FITC conjugate and incubated at 37°C for 30 min. After the reaction mixture was diluted with 1 mL PBST, the cells were recovered by centrifugation (14, 000 X *g* for 3 min) and washed once more with 1 mL deionized water. After the second centrifugation, most of the supernatant was aspirated, and the cell pellet was suspended in ~100 µL of the residual water. A 2-µL volume of the suspension was transferred to one well of a 12-well Teflon-coated microscope slide (Cel-Line/Erie Scientific Co., Portmouth, NH), air-dried, and mounted with DAKO faramount aqueous medium (DAKO Co., Carpinteria, CA). The labeled cells were visualized on a UV microscope with a 40X or 100X objective with oil. When *B. anthracis* cells exhibited whole-body bright green fluorescence against a dark background, the reaction was read as positive. A negative reaction had cells that did not show fluorescence. An identical procedure was used to stain 45-µL volumes of the processed clinical specimens. DFA was reported as positive for *B. anthracis* only when both CW- and CAP-DFA assays were positive.

To determine the lower limits of detection for both CW- and CAP-DFA assays, serial 10-fold dilutions (10^7^–10^3^ cells/mL) of the fresh cells of the control strains (Pasteur strain and *B. cereus*) were prepared, and 45-µL volumes of cell suspension were used as described.

## Results

Of 230 *B. anthracis* isolates analyzed, 228 (99%) were positive in the CW-DFA (Table 1) (19). Two isolates (one environmental isolate from a mill and one from a cow) that were negative by the CW-DFA assay were collected in Alabama in the 1950s (20). Among the non–*B. anthracis* isolates, 10 (43%) of 23 *B. cereus* and 2 (16.7%) of 12 *B. thuringiensis* were also CW-DFA positive. All 9 *B. subtilis,* 11 *B. megaterium,* and 1 *B. mycoides* strains were negative (Table 2). In all the positive reactions, >99% of the *B. anthracis* cells expressed cell wall polysaccharide antigen so that characteristic chain-forming rods were visualized with bright fluorescence (Figure, panel A). All the 149 *B. anthracis* isolates from the 2001 anthrax outbreak investigation were positive (Table 1). The sensitivity and specificity of the CW-DFA assay were 99% (228/230, 95% confidence intervals [CI]) and 78.6% (44/56, 95% CI), respectively.

**Table 1:** Origin, designations, and results of cell wall and capsule direct fluorescent-antibody assays for 230 *Bacillus anthracis* isolates^a^

Origin	No. of isolates	Temporal range and geographic origin	MLVA genotypes represented^b^	CW-DFA (% positive)	CAP-DFA (% positive)
Human isolates	31	1943–1997; Africa, Asia, Australia, Europe, North America	3,4,22,23,28,32,34, 35,36,37,41,43,44, 45,50,66,68	31 (100)	31 (100)
Animal isolates	29	1939–1997; Africa, Asia, Australia, Europe, North and South America	3,10,20,26,29,30,35,38,40,45,48,49,51, 55,57,78,80,81,84, 85,87, 89	29 (100)	29 (100)
Environmental isolates (e.g., soil, burial sites, wool, tannery, mill)	16	1950–1993; Africa, Asia, Europe, and North America	13,14,21,24,47,62, 69,73,77,79,82	15 (94)	16 (100)
pX01 plasmid cured	4	1950–1974; North America		3 (75)	4 (100)
pX02 plasmid cured	1	Africa		1 (100)	0 (0)
2001 anthrax outbreak	149	October 2001; United States	62	149 (100)	149 (100)
Total	230			228 (99)	229 (99.6)

^a^DFA, direct fluorescent antibody assay; CW, cell wall; CAP, capsule; MLVA, multiple-locus variable-number tandem repeat analysis. ^b^Keim P, et al. J Bacteriol 2000;182:2928–36 (19).

**Table 2:** Results of cell wall and capsule direct fluorescent-antibody assays for 56 strains of five *Bacillus* species^a^

Species	No. of strains	CW-DFA (% positive)	CAP-DFA (% positive)
*B. cereus*	23	10 (43)	0 (0)
*B. thuringiensis*	12	2 (17)	0 (0)
*B. megaterium*	11	0 (0)	1 (11)
*B. mycoides*	1	0 (0)	0 (0)
*B. subtilis*	9	0 (0)	0 (0)
Total	56	12 (21)	1 (1.7)

^a^DFA, direct fluorescent-antibody assay; CW, cell wall; CAP, capsule.

**Figure:**
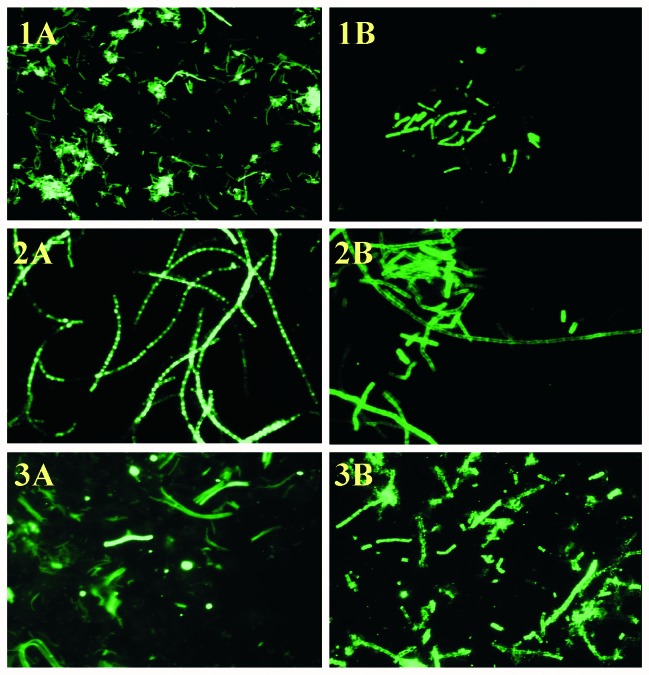
Direct fluorescent-antibody (DFA) staining of *Bacillus anthracis* cells. Panel A (cell wall DFA) and Panel B (capsule DFA) correspond to 1) Positive control (*B. anthracis* Pasteur strain), 2) Test isolate #2002013601 (environmental specimen, 2001 U.S. anthrax outbreak), and 3) Clinical specimen #2002007069 (lung tissue of patient 1, 2001 U.S. anthrax outbreak), original magnification x 400.

All but 1 (99.7%) of the 230 *B. anthracis* isolates tested were CAP-DFA positive; the single exception was a *B. anthracis* Sterne strain cured of plasmid pXO2 (Table 1) and, thus, as expected, it was unencapsulated. Of the 56 non–*B. anthracis* isolates tested, only 1 *B. megaterium* strain was positive by CAP-DFA assay (Table 2). This environmental isolate, collected during the bioterrorism-associated anthrax outbreak, was identified as *B. megaterium* by both standard microbiologic procedures (7) and sequencing of the 16S ribosomal RNA gene (data not shown). All the 149 *B. anthracis* isolates from the 2001 anthrax outbreak were CAP-DFA positive. Most of the encapsulated *B. anthracis* cells (>90%) were labeled uniformly (Figure, panel B), and they demonstrated similar fluorescence to that of the cell wall staining. The sensitivity and specificity of the CAP-DFA assay were 99% (229/230, 98% to 100% CI) and 98% (55/56, 90% to 100% CI), respectively.

Analysis of the combined DFA assay results showed that 227 of 230 *B. anthracis* isolates were positive, yielding a specificity of 99% (95% CI, 96% to 100%). Similarly, all 56 of the other *Bacillus* strains were negative, for a specificity of 100% (95% CI, 94% to 100%). The current two-component DFA assay was capable of detecting as low as ~10^4^ cells/mL of vegetative or encapsulated *B. anthracis* cells from cultures.

 Fourteen of the 26 clinical specimens analyzed from seven patients with laboratory-confirmed anthrax were positive in both the CW- and CAP-DFA assays (Table 3). Furthermore, most blood specimens (8 of 12) were positive by both assays. Most blood specimens were also positive by culture (n=5) and polymerase chain reaction (PCR) (n=4) assays. Among the other clinical specimens tested, two lung tissues, one lymph node, two pleural fluids, and one unspecified body fluid were positive by both DFA assays. Four of these six specimens were negative by culture, and three of them were positive by PCR. Most of these specimens were collected from patients treated with antimicrobial agents before or on the day of specimen collection. All other clinical specimens, such as heart fluid, pericardial fluid, and chest fluids, were negative by both DFA assays.

**Table 3:** Results of 26 clinical specimens from seven inhalational anthrax patients analyzed by direct fluorescent-antibody assay, culture, and polymerase chain reaction assay^a^

Patient identifier^b^	Specimen			Results		
	Type	Number	Date collected	DFA	Culture	PCR^c^
1	Heart blood^d,e^	1	10/6	(-)	ND	(-)
1	Blood^d,e^	1	10/6	(-)	ND	(-)
1	Lung tissue^d,e^	2	10/6	(+)	ND	(-)
1	Chest fluid^d,e^	2	10/6	(-)	ND	(+)
1	Pericardial fluid^d,e^	1	10/6	(-)	ND	(+)
2	Blood^d^	3	10/5	(-)	(-)	(-)
2	Pleural fluid^d^	1	10/5	(+)	(-)	(+)
2	Pleural fluid^d^	1	10/5	(-)	(-)	(+)
2	Unspecified body fluid^d^	1	10/5	(+)	(-)	(-)
3	Blood^d^	1	10/19	(+)	(-)	(+)
5	Blood^d^	2	10/21	(+)	(+)	(+)
6	Blood	1	10/22	(+)	(+)	(+)
10	Lung tissue^d,e^	1	10/31	(-)	(-)	(+)
10	Lymph node^d,e^	1	10/31	(-)	(-)	(+)
10	Pleural fluid^d^	1	10/29	(+)	(-)	(+)
10	Pleural fluid^d^	1	10/29	(-)	(-)	(+)
11	Blood	2	11/17	(+)	(+)	ND
11	Blood	2	11/17	(+)	(+)	ND
11	Lymph node^d-f^	1	11/21	(+)	(-)	(+)

^a^DFA, direct fluorescent-antibody assay; PCR, polymerase chain reaction; ND, not done. ^b^Patients 1, 2, 3, 5, 6, 10 reported by Jernigan et al*.* (6), and patient 11 reported by Barakat et al. (18). ^c^Real-time PCR as described by Hoffmaster et al. (20). All DNA samples tested positive by human beta actin PCR. ^d^Specimens collected the day on or after antimicrobial treatment was begun. ^e^Specimens collected postmortem. ^f^Documented culture negative; previously reported as culture positive (18).

## Discussion

 Recent events have emphasized the need for rapid, sensitive, and specific assays for the confirmatory identification of *B. anthracis* and detection of this agent directly in clinical specimens. The availability of monoclonal antibodies recognizing the cell-wall polysaccharide and capsule antigens of vegetative cells provides the means to rapidly differentiate *B*. *anthracis* from other *Bacillus* spp. Although some *B*. *cereus* and *B*. *thuringiensis* strains express the galactose/N-acetylglucosamine polysaccharide antigen, such organisms lack the poly-D-glutamic acid capsule of *B*. *anthracis.* Thus, detection of both antigens by a DFA assay is highly specific for *B. anthracis*. In this study, we evaluated a two-component DFA assay employing monoclonal antibodies specific for these two antigens for confirmatory identification of diverse *B*. *anthracis* strains and for detection of *B*. *anthracis* directly in clinical specimens. We found that this approach provided sensitive and specific confirmation of *B*. *anthracis* cultures within 3–6 h. In addition, this approach detected *B*. *anthracis* directly in clinical specimens of seven patients with laboratory-confirmed inhalational anthrax.

 The expression of DFA targets could vary by *B*. *anthracis* strain, which would adversely affect the sensitivity of the test. Consequently, we first evaluated the sensitivity of the two DFA assays independently against 230 *B*. *anthracis* isolates. Because of the diversity of *B*. *anthracis* isolates tested, our results should be applicable to very divergent strains from different sources. The sensitivity for *B*. *anthracis* was high (99%) for each DFA. The CW-DFA assay failed to detect only two isolates, and the CAP-DFA assay was negative only for the strain cured of the pXO2 plasmid, rendering it unencapsulated. This level of specificity of this two-component DFA assay was affirmed, as every outbreak-associated *B. anthracis* isolate tested positive. We determined that the minimal number of CFU detectable by either assay was 10^4^ CFU/mL, a level comparable with that of many PCR assays.

 The lower limit of detection is not a limiting parameter of the confirmatory test’s sensitivity because unlimited quantities of cells are available for testing after primary culture. However, specificity is crucial; CAP-DFA assay specificity was very high (98%), but the cell-wall assay specificity was only 78.6% compared with the previous studies on the limited cross-reactivity with the other *Bacillus* spp. (12). Almost 93% of the CW-DFA assay false-positive isolates were *B*. *cereus* or *B*. *thuringiensis.* Only one *B*. *megaterium* strain was CAP-DFA positive. However, confirmation of *B*. *anthracis* requires that both assays be positive; compliance with that requirement resulted in 100% specificity because no test isolate except *B*. *anthracis* was positive in both assays. Again, the high specificity of the two-component DFA assay was reflected in its performance on the 149 tested isolates from the 2001 anthrax outbreak. These isolates were shown to be indistinguishable from each other based on the molecular analysis, as delineated by Hoffmaster et al. (20). The DFA assay specificity was similar to the highest levels achieved by PCR assays and the phenotypic confirmatory identification scheme described previously (5,7). However, the two-component DFA assay requires less sophisticated equipment, reagents, and controls and smaller dedicated space than PCR, and is only slightly less rapid. The DFA assay is considerably more rapid than the standard confirmatory identification methods and offers a substantial specificity advantage.

 The availability of clinical material from several anthrax patients from the 2001 outbreak provided an additional opportunity to evaluate this two-component DFA. We used the DFA assay to detect *B*. *anthracis* directly in the limited number of available clinical specimens and compared these results with those from culture and PCR. We noted that all DFA-positive specimens reacted with both components of the assay, suggesting that the sensitivities and specificities of the respective assays were similar, as we previously showed for cultures. The two-component assay detected *B*. *anthracis* in all specimens that were positive by culture and the confirmatory identification regimen. Moreover, four of the five culture-negative specimens that were positive by DFA assay were also positive by PCR. The fifth such specimen (patient 2, unspecified fluid) and two additional specimens (patient 1, lung tissues) that were not cultured were positive only by the DFA assay. Four other specimens from these two patients were PCR positive, suggesting that the discordant DFA assay results were true positives. None of the specimens collected after the patient received antimicrobial therapy were culture positive, but four specimens collected from four patients concurrent with (n=1) or after (n=3) antimicrobial therapy were DFA positive. Together, these results suggest that the DFA assay is specific for *B*. *anthracis* and that its sensitivity is similar to that of culture or perhaps considerably greater if the patient is receiving antimicrobial agents. Conversely, six PCR-positive specimens were negative by the DFA assay, indicating that the latter may be relatively less sensitive. The two DFA assay–positive/PCR-negative specimens indicate that only performing all available assays on specimens may maximize diagnostic sensitivity. The two-component DFA assay rapidly detected *B*. *anthracis* in all seven anthrax patients, suggesting that its predictive value may have diagnostic relevance. However, the numbers of specimens and patients in this evaluation were limited.

 DFA assays have traditionally been used to rapidly identify bacterial cultures and to directly detect bacterial disease agents in infected clinical specimens. The extensive use of such assays depends on their ability to sensitively and specifically detect target organisms and to predict the diseases they cause. We report for the first time an evaluation of a two-component DFA assay to confirm the identity of presumptive *B*. *anthracis* cultures and to detect this agent in clinical specimens. The current assay had excellent sensitivity and specificity as a rapid confirmatory test for *B*. *anthracis* cultures performed in a real-time fashion in an outbreak setting. The assay also detected *B*. *anthracis* in a limited number of specimens from anthrax patients. However, we recommend that this latter application be limited to a presumptive role in the laboratory diagnosis of anthrax, until positive and negative predictive values are better defined by future evaluations in animal models and human populations with high anthrax prevalence or outbreaks.

## References

[1] Pile J, Malone J, Eitzen E, Friedlander A. Anthrax as a potential biological warfare agent. Arch Intern Med. 1998;158:429–34. 10.1001/archinte.158.5.429 9508220

[2] Inglesby T, Henderson D, Bartlett J, Ascher M, Eitzen E, Friedlander A, Anthrax as a biological weapon. JAMA. 1999;281:1735–45. 10.1001/jama.281.18.1735 10328075

[3] Christopher G, Cieslak T, Pavlin A, Eitzen E. Biological warfare. A historical perspective. JAMA. 1997;278:412–7. 10.1001/jama.278.5.412 9244333

[4] Kliemann W, Ruoff K. Bioterrorism: implications for the clinical microbiologist. Clin Microbiol Rev. 2001;14:364–81. 10.1128/CMR.14.2.364-381.2001 11292643 PMC88979

[5] Khan A, Morse S, Lillibridge S. Public-health preparedness for biological terrorism in the USA. Lancet. 2000;356:1179–82. 10.1016/S0140-6736(00)02769-0 11030310

[6] Jernigan J, Stephens D, Ashford D, Omenaca C, Topiel M, Galbraith M, Bioterrorism-related inhalational anthrax: the first 10 cases reported in the United States. Emerg Infect Dis. 2001;7:933–48. 11747719 10.3201/eid0706.010604 PMC2631903

[7] Isenberg H. Clinical microbiology procedures handbook. vol.1 & 2. Washington: American Society for Microbiology Press; 1992.

[8] Phillips A, Ezzell J. Identification of *Bacillus anthracis* by polyclonal antibodies against extracted vegetative cell antigens. J Appl Bacteriol. 1989;66:419–32. 2502530 10.1111/j.1365-2672.1989.tb05111.x

[9] Franek J. Application of fluorescent antibodies for demonstrating *B. anthracis* in the organs of infected animals. J Hyg Epidemiol Microbiol Immunol. 1964;8:111–9. 14128970

[10] Helgason E, Okstad D, Caugant H, Johansen A, Fouet M, Mock M, *Bacillus anthracis, Bacillus cereus,* and *Bacillus thuringiensis*—one species on the basis of genetic evidence. Appl Environ Microbiol. 2000;66:2627–30. 10.1128/AEM.66.6.2627-2630.2000 10831447 PMC110590

[11] Ezzell J, Abshire T. Immunological analysis of cell-associated antigens of *Bacillus anthracis.* Infect Immun. 1988;56:349–56. 3123387 10.1128/iai.56.2.349-356.1988 PMC259287

[12] Ezzell J, Abshire T, Little S, Lidgerding B, Brown C. Identification of *Bacillus anthracis* by using monoclonal antibody to cell wall galactose-N-acetylglucosamine polysaccharide. J Clin Microbiol. 1990;28:223–31. 2107201 10.1128/jcm.28.2.223-231.1990 PMC269580

[13] Fouet A, Mesnage S, Tosi-Couture E, Gounon P, Mock M. *Bacillus anthracis* surface: capsule and S-layer. J Appl Microbiol. 1999;87:251–5. 10.1046/j.1365-2672.1999.00882.x 10475960

[14] Etienne-Toumelin I, Sirard J, Duflot E, Mock M, Fouet A. Characterization of the *Bacillus anthracis* S-layer: cloning and sequencing of the structural gene. J Bacteriol. 1995;177:614–20. 7836294 10.1128/jb.177.3.614-620.1995 PMC176635

[15] Ezzell J, Welkos S. The capsule of *Bacillus anthracis*, a review. J Appl Microbiol. 1999;87:250. 10.1046/j.1365-2672.1999.00881.x 10475959

[16] Ezzell J, Abshire T. Encapsulation of *Bacillus anthracis* spores and spore identification. Proceedings of the International Workshop on Anthrax. Salisbury Medical Bulletin 1999:87:42.

[17] Mina B, Dym F, Kuepper F, Tso R, Arrastia C, Kaplounova I, Fatal inhalational anthrax with unknown source of exposure in a 61-year-old woman in New York City. JAMA. 2002;287:858–62. 10.1001/jama.287.7.858 11851577

[18] Barakat L, Quentzel H, Jernigan J, Kirschke D, Griffith K, Spear S, Fatal inhalational anthrax in a 94-year-old Connecticut women. JAMA. 2002;287:863–8. 10.1001/jama.287.7.863 11851578

[19] Keim P, Price L, Klevytska A, Smith K, Schupp J, Okinaka R, Multi-locus variable-number tandem repeat analysis reveals genetic relationships with *Bacillus anthracis.* J Bacteriol. 2000;182:2928–36. 10.1128/JB.182.10.2928-2936.2000 10781564 PMC102004

[20] Hoffmaster A, Meyer R, Bowen M, Marston C, Weyant R, Barnett G, Evaluation and validation of a real-timepolymerase chain reaction assay for rapid identification of *Bacillus anthracis.* Emerg Infect Dis. 2002;8:1178–82. 12396935 10.3201/eid0810.020393 PMC2730313

